# Leveraging Automated Machine Learning for Environmental Data‐Driven Genetic Analysis and Genomic Prediction in Maize Hybrids

**DOI:** 10.1002/advs.202412423

**Published:** 2025-03-06

**Authors:** Kunhui He, Tingxi Yu, Shang Gao, Shoukun Chen, Liang Li, Xuecai Zhang, Changling Huang, Yunbi Xu, Jiankang Wang, Boddupalli M. Prasanna, Sarah Hearne, Xinhai Li, Huihui Li

**Affiliations:** ^1^ State Key Laboratory of Crop Gene Resources and Breeding Institute of Crop Sciences Chinese Academy of Agricultural Sciences (CAAS) CIMMYT‐China Office Beijing 100081 China; ^2^ Nanfan Research Institute CAAS Sanya Hainan 572024 China; ^3^ International Maize and Wheat Improvement Center (CIMMYT) Apdo. Postal 6‐641 Texcoco D.F. 06600 Mexico; ^4^ CIMMYT International Centre for Research in Agroforestry (ICRAF) House Nairobi 00100 Kenya

**Keywords:** environmental data, genetic analysis, genomic selection, genotype‐by‐environment interactions, machine learning

## Abstract

Genotype, environment, and genotype‐by‐environment (G×E) interactions play a critical role in shaping crop phenotypes. Here, a large‐scale, multi‐environment hybrid maize dataset is used to construct and validate an automated machine learning framework that integrates environmental and genomic data for improved accuracy and efficiency in genetic analyses and genomic predictions. Dimensionality‐reduced environmental parameters (RD_EPs) aligned with developmental stages are applied to establish linear relationships between RD_EPs and traits to assess the influence of environment on phenotype. Genome‐wide association study identifies 539 phenotypic plasticity trait‐associated markers (PP‐TAMs), 223 environmental stability TAMs (Main‐TAMs), and 92 G×E‐TAMs, revealing distinct genetic bases for PP and G×E interactions. Training genomic prediction models with both TAMs and RD_EPs increase prediction accuracy by 14.02% to 28.42% over that of genome‐wide marker approaches. These results demonstrate the potential of utilizing environmental data for improving genetic analysis and genomic selection, offering a scalable approach for developing climate‐adaptive maize varieties.

## Graphical abstract

An automated machine learning framework integrating environmental and genomic data enhances genetic analysis and genomic prediction in maize. By leveraging dimension‐reduced environmental parameters, it reveals trait‐environment relationships and identifies genetic markers that govern phenotypic plasticity and genotype‐by‐environment interactions. The combined use of markers and environmental features improves genomic prediction accuracy, offering a scalable solution for developing climate‐resilient maize varieties.

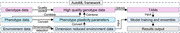

## 1 Introduction

The combination of genotype (G), environment (E), and their interaction determines plant phenotype and adaptability.^[^ ^1^, ^2^ ^]^ The phenotypic plasticity (PP) of a genotype describes its differential performance across environmental gradients,^[^ ^3^, ^4^ ^]^ and genotype‐environment interactions (G×E) therefore reflect variation in PP across different genotypes.^[^ ^5^, ^6^, ^7^ ^]^ As the adverse impacts of climate change on crop yield intensify, breeding climate‐resilient crop varieties becomes increasingly critical to ensuring global food security.^[^ ^8^, ^9^ ^]^ To this end, defining the genetic mechanisms of PP and G×E interactions is an essential step in cultivating climate adaptive crop varieties.

In the past 10 years, although some studies have successfully identified some loci associated with PP and G×E,^[^ ^10^, ^11^, ^12^, ^13^, ^14^, ^15^, ^16^ ^]^ these studies did not examine differences in the genetic basis of PP and G×E, nor do these reports clarify which environmental parameters (EPs) specifically affect such loci that interact with the environment. The recently developed Critical Environmental Regressor through Informed Search‐Joint Genomic Regression Analysis (CERIS‐JGRA) approach incorporates environmental data into statistical models to identify relationships between genetic and EPs,^[^ ^17^ ^]^ and has been applied to analyze the genetic basis of PP in crops such as rice, maize, wheat, sorghum, and oats, revealing key EPs that affect PP.^[^ ^18^, ^19^, ^20^, ^21^, ^22^ ^]^ However, this approach cannot detect PP‐related genetic loci relevant to specific developmental stages, which is also essential for a robust landscape perspective of the genetic basis of PP.

Alternatively, breeding can be accelerated through genomic selection,^[^ ^23^ ^]^ which largely shifts the decision‐making from human judgment to biological data‐driven statistical models, streamlining the breeding process and improving efficiency.^[^ ^24^, ^25^ ^]^ Some studies have tested complex trait genetic loci for model training instead of whole‐genome genetic markers, which proved effective for enhancing model performance and enabled the application of complex trait genetic loci in breeding.^[^ ^26^, ^27^, ^28^ ^]^ By contrast, environment‐responsive genetic loci have yet to be explored for enhancing model performance. In addition, some studies have begun integrating environmental data into predictive models,^[^ ^29^, ^30^, ^31^, ^32^ ^]^ as EPs affecting phenotype could potentially improve prediction accuracy and enable joint multi‐environment phenotype predictions about crop adaptability to new or changing environments. We therefore speculate that applying environment‐responsive genetic loci and EPs in genomic prediction may enhance performance in modeling traits across environments.

Historically, most models have used reaction norm models to account for changes in genotype performance across trials as a function of measurable characteristics of those trials, called environmental covariates (ECs).^[^ ^21^, ^33^, ^34^ ^]^ As potential ECs have high dimensionality, statistical models employing ECs must operate robustly in high‐dimensional spaces, posing a significant challenge to the model's intrinsic capabilities. Machine learning (ML) has opened new opportunities for genomic selection‐based breeding,^[^ ^35^, ^36^ ^]^ which provides several advantages, including computational efficiency, high data handling capacity, and the ability to integrate multidimensional data.^[^ ^35^, ^37^, ^38^ ^]^ ML can also capture complex nonlinear relationships between features to enhance predictive performance of models.^[^ ^39^, ^40^ ^]^ Therefore, we hypothesized that ML could help overcome challenges in incorporating environmental data and multi‐environment joint prediction into genomic prediction tasks.

To explore this possibility in the current study, we applied an automated machine learning (AutoML) framework incorporating environmental data in genetic analysis and genomic predictions of a public dataset comprising 1000 maize hybrids across seven experimental environments, with supplementary validation in additional datasets from 2808 maize hybrids and 286 wheat lines. We reduced the dimension of EPs based on the developmental stages of maize hybrids, then screened for genetic loci involved in PP and G×E using genome‐wide association study (GWAS), and compared differences in these loci between PP and G×E. We found that integrating GWAS loci and dimensionality‐reduced environmental parameters (RD_EPs) into genomic prediction models resulted in higher prediction accuracy than other statistical models in different prediction tasks, and showed good scalability in independent test datasets. Collectively, our study serves as a reference pipeline for genetic loci involved in PP and G×E interactions that guide the incorporation of environmental data into genomic selection.

## 2 Results

### 2.1 Phenotypic Plasticity and Genotype‐Environment Interactions Influencing Agronomic and Yield Traits in Maize Hybrids

**Figure 1:**
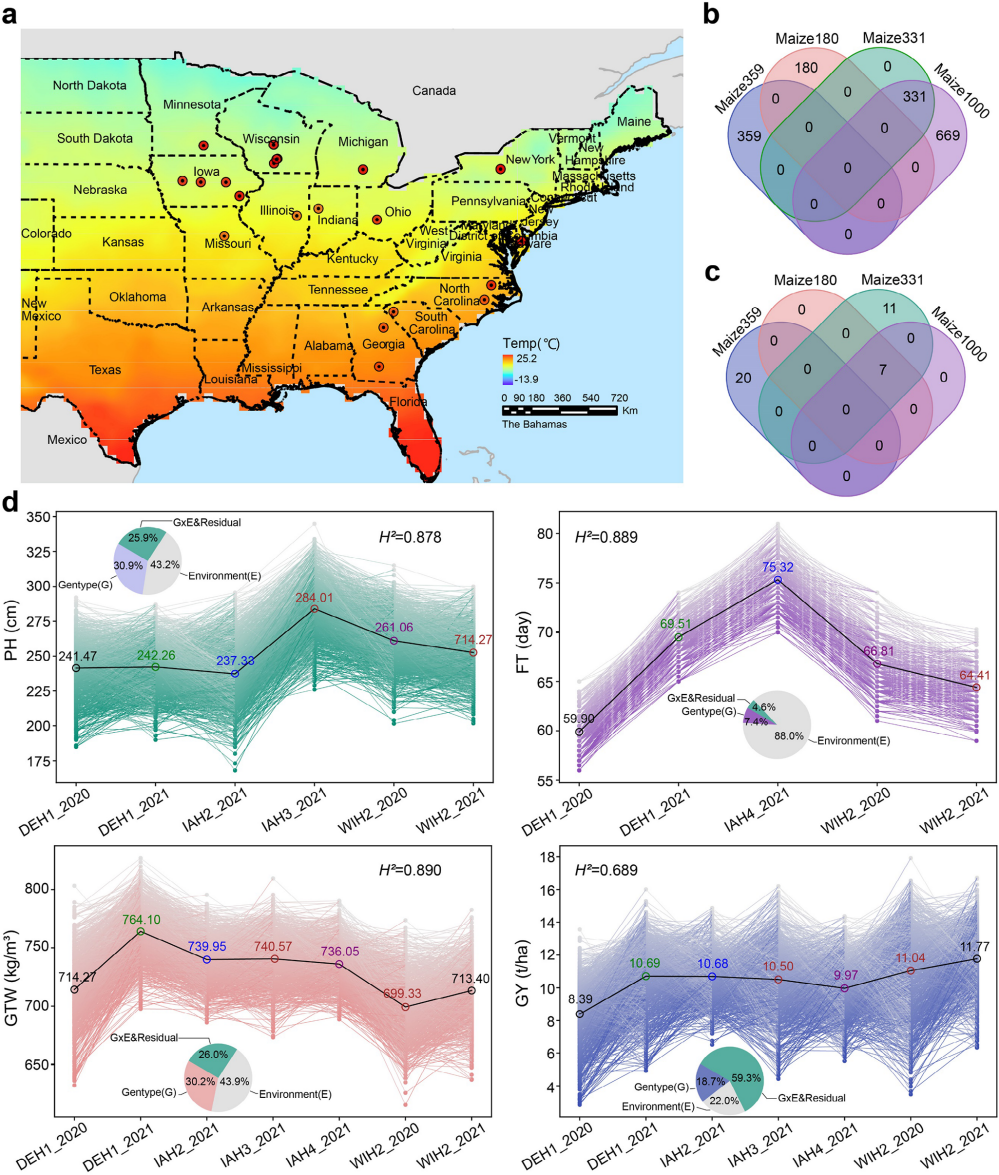
Overview of multi‐environment trials for maize hybrids. a) Geographical distribution of 36 experimental environments (year‐location combinations) in the United States, which were distributed in 21 locations. b) The overlap in the distribution of maize hybrids among the Maize1000, Maize180, Maize331 and Maize359 datasets. c) The overlap in cultivation environments among the Maize1000, Maize180, Maize331 and Maize359 datasets. d) Phenotypic variability of each hybrid in the Maize1000 dataset for plant height (PH), flowering time (FT), grain test weight (GTW), and grain yield (GY) responding to environments. The black lines connect the mean phenotypes across different environments, with circle and numerical values representing the mean phenotypes. The pie charts show the proportion of different variance components contributing to the total phenotypic variance.

We obtained the publicly available Genomes to Fields (G2F) dataset,^[^ ^41^, ^42^ ^]^ which included 1539 maize hybrids cultivated across 21 locations in the eastern and midwest United States (**Figure** **1**a), and spanning three growing seasons (2020, 2021, and 2022), resulting in 36 total environments (i.e., year‐location combinations). The traits of interest included plant height (PH), flowering time (FT), grain test weight (GTW), and grain yield (GY). The 1539 hybrids were divided into four genotype datasets: Maize1000, Maize180, Maize331, and Maize359. The relationships between the genotypes and environments in these four datasets are illustrated in Figure 1b,c. Figure 1b shows overlap in the distribution of maize hybrids among the four genotype classes; Figure 1b shows overlap in cultivation environments among the four genotype classes. Although Maize331 is a subset of the Maize1000 genotype dataset, the other genotypes shared no overlap among datasets. Maize180 and Maize1000 phenotyping was conducted in the same environments, Maize331 and Maize1000 were phenotyped in some overlapping environments, while Maize359 was phenotyped in independent environments. Details of experimental environments for each dataset are provided in Table S1 (Supporting Information).

In the Maize1000 dataset, within population comparisons showed significant phenotypic variation for each trait, with variation levels differing among environments and displaying obvious G×E effects. PP patterns also showed marked differences in variability among traits. For example, PP could be observed in FT across environments, but all individuals followed a similar trend, suggesting a weak influence of G×E interactions. In contrast, PP followed complex patterns for PH, GTW, and GY between environments, with frequent G×E interactions (Figure 1d). To quantify the effect of these interactions with environment, we analyzed the contribution of G, E, and G×E interactions (including residual variance) to each phenotype.

This analysis revealed that G, E, and G×E exerted differing influences on each of the four traits (Figure 1d), with the most profound effects observed in the influence of E on FT (i.e., accounting for 88.0% of the phenotypic variance) and G×E contributing 59.3% of variance in GY. By contrast, G accounted for 30.9% and 30.2%, E for 43.2% and 43.9%, and G×E for 25.9% and 26.0% of the phenotypic variance in PH and GTW, respectively. These results indicated that E primarily influences FT, while G, E, and G×E can each individually impact PH and GTW, and GY is most strongly affected by G×E. The heritability of PH was 0.878, 0.889 for FT, 0.890 for GTW, and 0.689 for GY (Figure 1d), indicating these phenotypes were largely reproducible across environments. These results collectively demonstrated the strong influence of E and G×E on the phenotype of maize hybrids, underscoring the importance of considering environmental data in genomic predictions and investigations of the genetic basis of maize traits.

### 2.2 An Automated Machine Learning Framework for Genome‐Wide Association Study and Genomic Prediction of Maize Hybrid Phenotypes

**Figure 2:**
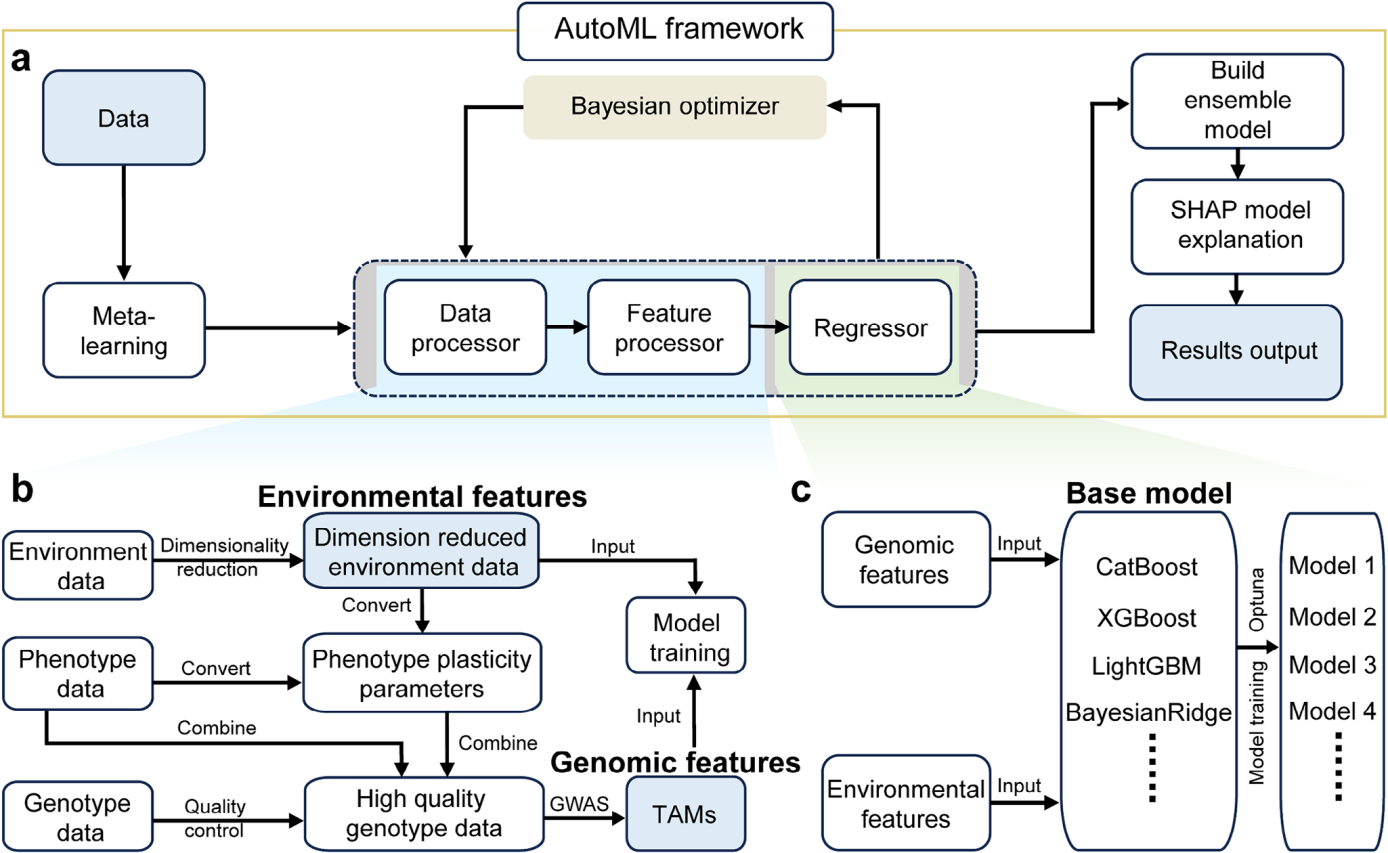
Design of automated machine learning framework for genetic analysis and genomic prediction in maize hybrids. a) Workflow of automated machine learning in genomic prediction. b) Detailed process of data and feature processing in automated machine learning (AutoML) framework. GWAS: genome‐wide association study; TAMs: trait‐associated markers. c) Base model training procedure in AutoML framework.

To facilitate incorporating environmental data into genetic analysis and genomic prediction, we designed an AutoML framework integrating data processing, environmental feature handling, GWAS, model training, and phenotypic prediction functions (**Figure** **2**a). The data processing and feature handling functions included genotype quality control, dimensionality reduction of EPs, and calculation of PP parameters (Figure 2b). The three‐variance‐component mixed model (3VmrMLM) method^[^ ^43^ ^]^ was used for GWAS to identify trait‐associated markers (TAMs) associated with specific traits, then apply those TAMs as genetic features along with RD_EPs as environmental features in model training. The model training function integrates a variety of base machine learning models, with the Optuna automated hyperparameter tuning algorithm^[^ ^44^ ^]^ to optimize the model parameters, a stacking algorithm^[^ ^45^ ^]^ for model ensembling to improve predictive accuracy, and SHapley Additive exPlanations (SHAP)^[^ ^46^ ^]^ to enhance model interpretability (Figure 2c). A phenotype prediction function facilitates validation with independent test data, including predictions for untested genotypes in a tested environment, tested genotypes in untested environments, and untested genotypes in untested environments. Pearson correlation coefficient (PCC) was used to evaluate the predictive accuracy of the final model.

### 2.3 Environmental Parameters Associated with Maize Developmental Stages and Phenotypic Plasticity for Genome‐Wide Association Study

**Figure 3:**
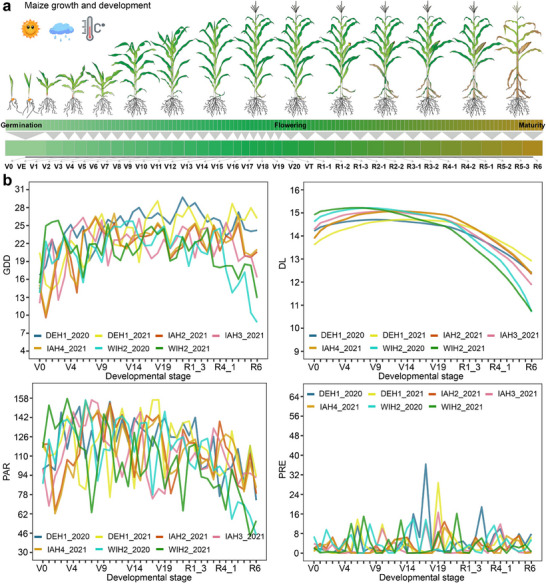
Dimensionality reduction of environmental parameters according to the development period of maize hybrids. a) The 36 development stage‐environment windows (V0 to R6) for maize hybrids were defined based on the relationship between the developmental stages and growing degree days (GDD). b) Trends in GDD, day length (DL), photosynthetically active radiation (PAR), and precipitation (PRE) across 36 development stage‐environment windows.

Growth and development are strongly influenced by EPs in maize, and in particular, accumulated temperature (i.e., growing degree days, GDD) is highly correlated with development stage, with specific stages initiating after GDD reaches a favorable range (Table S1, Supporting Information). To reduce the dimensionality of EPs, development stage‐environment windows was established based on the relationship between the 36 development stages in maize hybrids (from sowing to maturity: V0‐R6) and GDD^[^ ^47^ ^]^ (**Figure** **3**a). We then determined the average EPs for day length (DL), GDD, precipitation (PRE), photosynthetically active radiation (PAR), air relative humidity (RH), photothermal time (PTT), photothermal ratio (PTR), daily diurnal temperature range (DTR), and photothermal sensitivity (PTS) corresponding to each window (Table S2, Supporting Information), resulting in a unique EP profile for each window (Figure 3b; Figure S2, Supporting Information). Plots of these parameters for each complete growing season showed considerable fluctuation, reflecting the complexity of environmental changes (Figure 3b; Figure S2, Supporting Information), which may be pivotal in determining the effects of PP and G×E interactions on traits.

**Figure 4:**
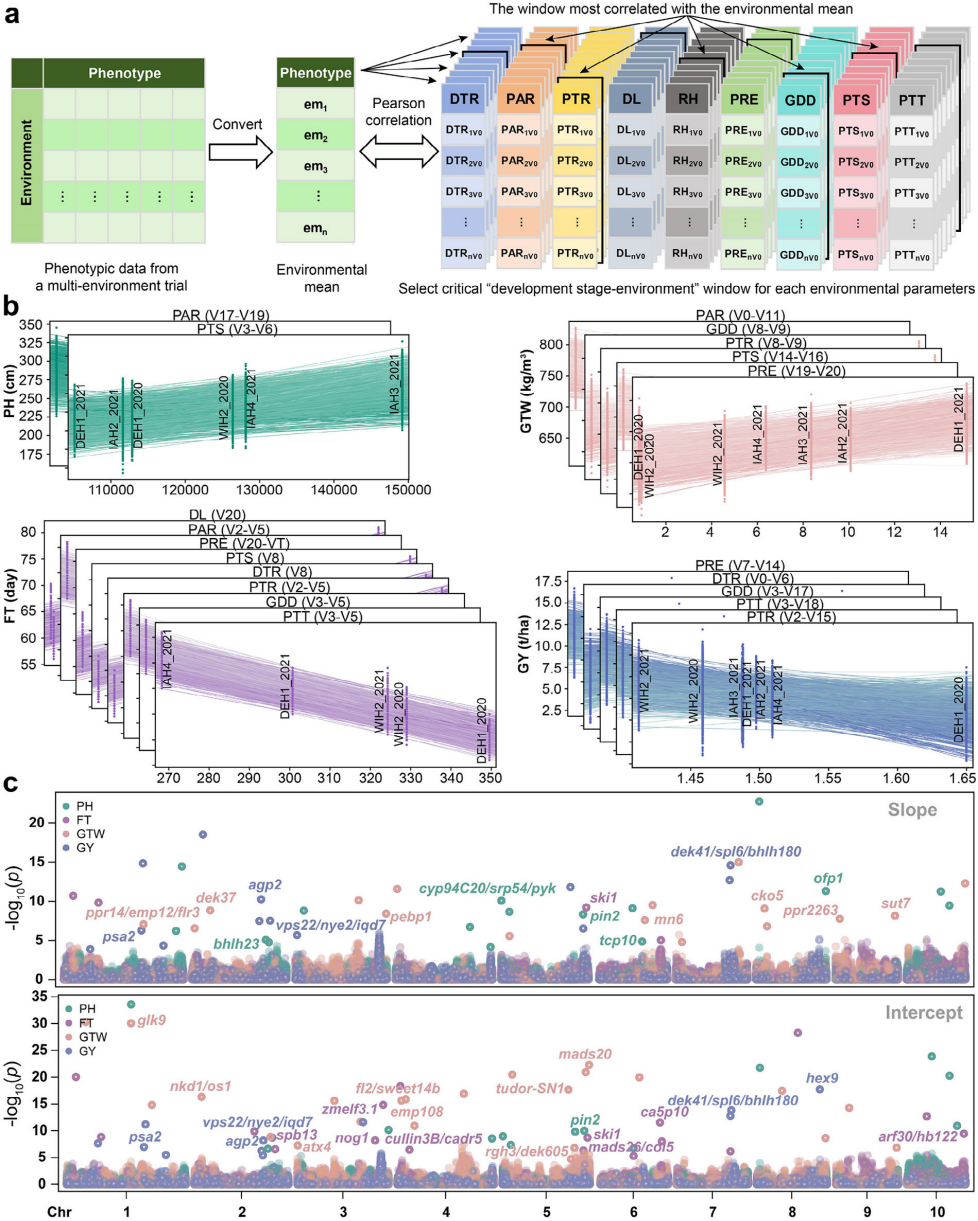
Phenotypic plasticity parameters estimation and genome‐wide association study. a) Flowchart for determining the critical development stage‐environment windows for each trait and dimensionality‐reduced environmental parameters (RD_EPs). DTR: daily diurnal temperature range; PAR: photosynthetically active radiation; PTR: photothermal rate; DL: day length; RH: air relative humidity; PRE: precipitation; GDD: growing degree days; PTS: photothermal sensitivity; PTT: photothermal time. b) Phenotypic plasticity (PP) parameters modeled by RD_EPs for PH, FT, GTW, and GY. Each dot denotes the observed phenotypic value for each genotype in an environment, and each line is the regression‐fitted value for each genotype. c) Manhattan plot of genome‐wide association study (GWAS) for PP. The slope and intercept represent PP trait‐associated markers (PP‐TAMs). The different colors of the dots in the plot represent different traits. PH: plant height; FT: flowering time; GTW: grain test weight; GY: grain yield.

To establish a relationship between EP and traits, we first calculated the phenotypic mean of four traits across each environment. Then, by setting different sliding window sizes, computed all possible values of the nine EPs within any development stage‐environment window in the range of V0 to R6. Pearson correlation analysis between the mean values for each trait and the nine EPs in each developmental window showed that PH was most strongly correlated with PTS in V3 to V6 development stage (*r* = 0.964); FT was most strongly correlated with PTT in V3 to V5 development stage; GTW was most strongly correlated with PTS in V14 to V16 (*r* = −0.953) and PRE in V19 to V20 (*r* = 0.953); GY was most strongly correlated with PTR in V2 to V15 (*r* = −0.986) (**Figure** **4**a; Figures S3a and S4, Supporting Information). These windows in which EPs were most strongly correlated with traits (*r* ≥ 0.90 or *r* ≤ −0.90) were subsequently designated critical development stage‐environment windows (referred to as critical windows hereafter). In these critical windows, we could observe clear linear effects of EPs on various traits during specific growth stages. For instance, FT was predominantly influenced by EPs in the early vegetative growth stage (DTR, V8; PAR, V2–V5; PTR, V2–V5; GDD, V3–V5; PTS, V8; PTT, V3–V5), whereas GY was affected by EPs in both the early and late vegetative growth stages (DTR, V0–V6; PTR, V2–V15; PRE, V7–V14; GDD, V3–V17; PTT, V3–V18). These findings underscored the significance of vegetative growth phase in determining the phenotype and yield of maize hybrids.

Regression analysis of the critical windows revealed a strong linear correlation between trait mean values and the nine EPs (critical windows) across environments (Figures S3b and S5, Supporting Information). A steeper slope in these regression analyses indicates greater phenotypic variability (i.e., higher PP, less environmental adaptability) and vice versa. We therefore assessed this linear relationship between each individual trait value and each EP for each critical window (Figure 4b). The resulting PP scores (intercept and slope) for each trait showed significant correlations between PH and 2 EPs, FT and 8 EPs, GTW and 5 EPs, and GY and 5 EPs (Figure 4b; Figure S3a, Supporting Information), which were subsequently used as PP parameters in GWAS.

### 2.4 Genome‐Wide Association Study Based on Phenotypic Plasticity and Genotype‐Environment Interaction

To dissect the genetic basis of PP and G×E, we first examined the distribution of trait values and PP parameters. We found that trait values in different environments followed a normal distribution, and slope was strongly correlated with intercept in fitting equations between EPs and traits, both of which followed a normal distribution (Figure S6 and Tables S3 and S4, Supporting Information), suggesting these traits and PP parameters were suitable for GWAS. We then analyzed single‐environment GWAS of PP parameters and joint GWAS across multiple environments for trait values to explore TAMs that influence PP and G×E interactions.

GWAS subsequently identified 854 total TAMs, including 539 PP‐TAMs (230 for slope and 309 for intercept), 223 environmental stability TAMs (Main‐TAMs), and 92 G×E‐TAMs. Since each trait corresponds to multiple PP parameters, GWAS detected many identical PP‐TAMs. After merging these identical PP‐TAMs, we found that 39 PP‐TAMs, 67 Main‐TAMs, and 24 G×E‐TAMs were associated with PH; 95, 47, and 16 with FT; 92, 67, and 28 GTW; and 57, 42, and 24 with GY, respectively (Figure 4c; Figure S7 and Table S5, Supporting Information). Although a multitude of common TAMs were identified across different PP parameters for each trait, a substantial number of PP parameter‐specific TAMs were still detected. For example, in PH, 43.10% of the TAMs detected using PP parameters derived from PTS and PAR were found to be nonoverlapping. These results highlighted the informative value of PP parameters derived from different EPs in genetic analysis, which can facilitate exploration of the genetic basis of phenotypic plasticity of target traits.

To validate the reliability of these TAMs, we selected TAMs for four traits based on their effect sizes, including three large‐effect, three moderate‐effect, and three small‐effect TAMs from each category (Main‐TAMs, G×E‐TAMs, Slope‐TAMs, and Intercept‐TAMs). Comparison of phenotypic variations among different genotypes (AA, Aa, and aa) across multiple environments identified significant phenotypic differences (*P* < 0.05) between the AA and aa allele combinations of all selected TAMs in at least two environmental conditions (Table S6, Supporting Information), thus confirming the reliability of these TAMs. We then searched for reported functional genes related to each target trait in the 1Mb chromosomal regions upstream and downstream of TAM (When *r* ^2^ = 0.2, the whole genome linkage disequilibrium (LD) decays to approximately 2Mb; Figure S11, Supporting Information). Approximately 42.5% of the TAMs were associated with genes of known function in maize or with orthologous genes of known function from other species in maize (Figure 4c; Figure S7 and Table S5, Supporting Information). Among them, 64, 61, 69, and 65 known functional genes or orthologous genes were respectively identified for PH, FT, GTW, and GY, including classic star genes such as *d1* (*Zm00001d039634*), *d9* (*Zm00001d013465*), *zfl1* (*Zm00001d026231*), *knr6* (*Zm00001d03662*), and *o11* (*Zm00001d03677*).

To compare the genetic basis of PP with that of G×E, we first defined the 1Mb chromosomal regions upstream and downstream of the TAMs as quantitative trait loci (QTLs) and merged overlapping QTLs into a single QTL to mitigate the impact of positional bias in TAMs caused by LD in subsequent analyses. Through this step, we obtained 20 G×E‐QTLs and 35 PP‐QTLs for PH, 15 and 83 for FT, 22 and 76 for GTW, and 21 and 48 for GY, respectively. We then examined the co‐localization of G×E‐QTLs and PP‐QTLs for each trait and found that in PH, five QTLs were co‐localized between PP and G×E, accounting for 14.3% of the total PP‐QTLs and 25.0% of the total G×E‐QTLs, respectively; in FT, four such co‐localized QTLs were identified, accounting for 4.8% of the total PP‐QTLs and 26.7% of the total G×E‐QTLs, respectively; in GTW, 13 co‐localized QTLs were identified, accounting for 17.1% of the total PP‐QTLs and 59.1% of the total G×E‐QTLs, respectively; and in GY, six co‐localized QTLs were detected, accounting for 12.5% of the total PP‐QTLs and 28.6% of the total G×E‐QTLs, respectively (Table S7, Supporting Information). These results suggested that the genetic loci underlying PP shared relatively little overlap with those loci mediating the effects of G×E interaction.

### 2.5 Using Trait‐Associated Markers and Dimensionality‐Reduced Environmental Parameters as Features to Enhance Accuracy of Genomic Prediction

To explore the value of TAMs and EPs in genomic prediction for breeding, we constructed an ensemble genomic prediction model including Categorical Boosting (CatBoost), Extreme Gradient Boosting (XGBoost), Light Gradient Boosting Machine (LightGBM), and BayesianRidge within the AutoML framework. We then utilized fivefold cross‐validation to partition the Maize1000 dataset into training and validation sets and evaluated the model's predictive accuracy for the four traits using the PCC between observed and predicted values.

To determine whether and how each of these base models contributed to prediction accuracy, we initially conducted ablation experiments to quantify their respective impacts on predictive performance of the ensemble model. Using All‐TAMs and DR_EPs as features, we compared the performance of single models, two‐model ensembles, three‐model ensembles, and the full four‐model ensemble. The results demonstrated that the ensemble model, including all four algorithms, consistently achieved the highest predictive accuracy across all traits (Figure S8, Supporting Information). For example, the four‐model ensemble achieved a predictive accuracy of 0.878 for PH, compared to 0.875 for the three‐model ensemble, 0.872 for the two‐model ensemble, and 0.853 for the single model (Figure S8a, Supporting Information). Similarly, the four‐model ensemble achieved a predictive accuracy of 0.619 for GY, while the three‐model, two‐model, and single‐model ensemble achieved predictive accuracy of 0.604, 0.602, and 0.590, respectively (Figure S8d, Supporting Information). These findings underscore the role of individual base models in enhancing the predictive accuracy of the ensemble model.

**Figure 5:**
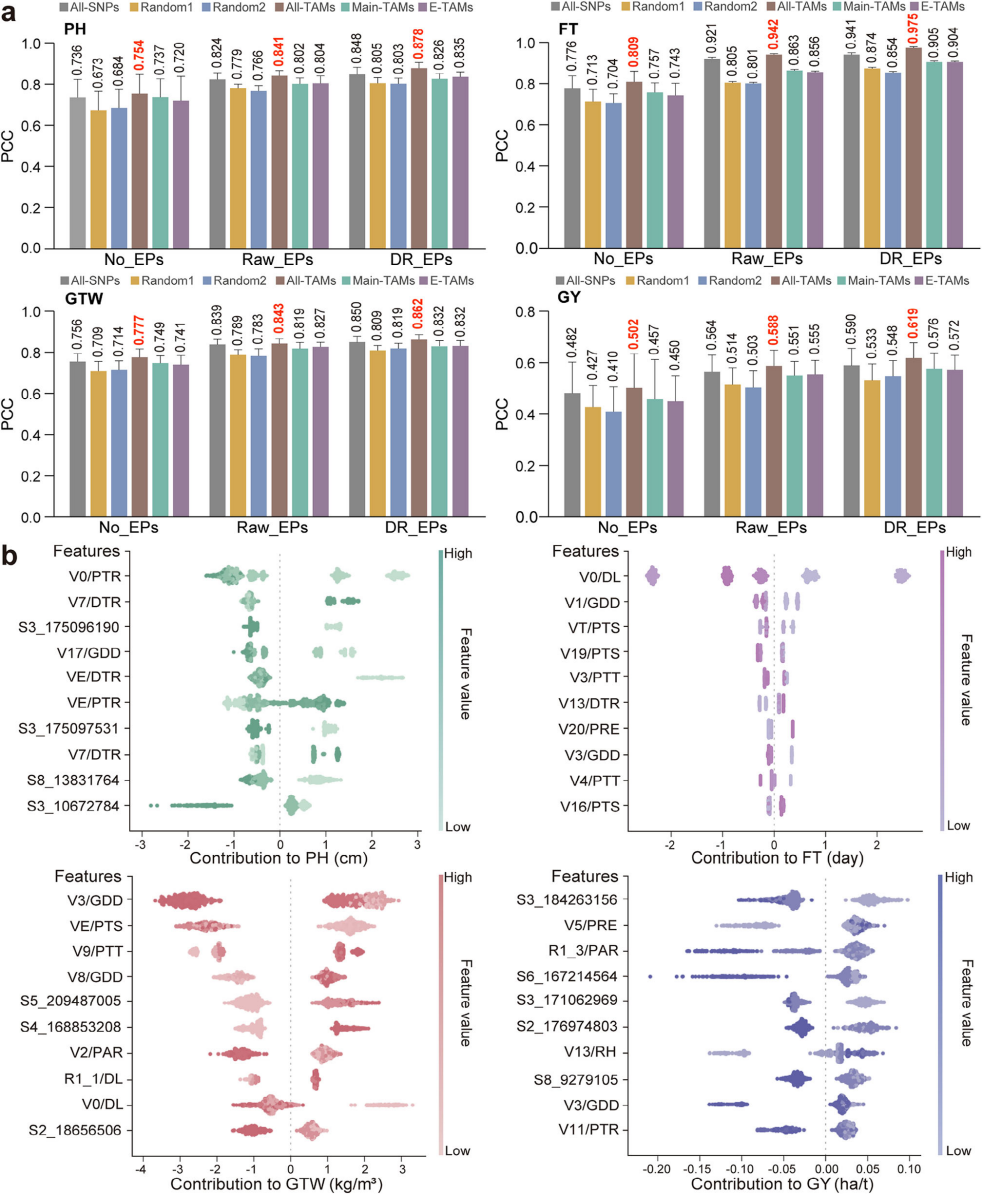
Train and interpret of genomic prediction models with automated machine learning framework. a) The impact of different combinations of genetic and environmental features on model prediction accuracy. No_EPs represent no EPs were used, Raw_EPs represent raw environmental parameters, and RD_EPs represent dimensionality‐reduced environmental parameters. All‐SNPs: Genome wide quantitative trait nucleotides without feature extraction. Main‐TAMs: environmental stability trait‐associated markers. E‐TAMs: Genotype‐environment interactions trait‐associated markers plus phenotypic plasticity trait‐associated markers. All‐TAMs: Main‐TAMs plus E‐TAMs. Random1: Based on the number of all TAMs detected by GWAS, randomly select SNPs from the genome, and then extract the loci within 37 kb upstream and downstream of the random SNPs as genetic features. Random2: Based on the number of All‐TAMs and all SNPs within the 37 kb interval upstream and downstream of the TAMs, randomly select the same number of SNPs from the genome as genetic features. b) Model interpretations. Shapley Additive Explanation (SHAP) values show the influence of each feature on the final phenotype prediction for each hybrid. A negative SHAP value suggests that a specific feature factor had a negative effect on the prediction, whereas a positive SHAP value indicates a positive effect on the prediction. PH: plant height; FT: flowering time; GTW: grain test weight; GY: grain yield.

Next, we explored the impact of various genetic and environmental features on model performance. The genetic features employed for model training included all single nucleotide polymorphisms (All‐SNPs), randomly selected SNPs (Random1 and Random2), All‐TAMs (including Main‐TAMs and environment‐responsive TAMs), Main‐TAMs, and environment‐responsive TAMs (E‐TAMs, including G×E‐TAMs and PP‐TAMs). The environmental features used in model training include both the raw EPs (Raw_EPs) and DR_EPs. Comparison of model performance for all traits using different combinations of these features revealed that, for all traits and ignoring EPs, using All‐TAMs specific to each trait as features resulted in better predictive accuracy than using All‐SNPs, Random1, Random2, or only the Main‐TAMs and E‐TAMs (**Figure** **5**a). Compared to using All‐SNPs, employing All‐TAMs resulted in average increases of 2.68%, 3.38%, 1.56%, and 4.44% in predictive accuracy for PH, FT, GTW, and GY, respectively. In comparison to using only Main‐TAMs, adding E‐TAMs led to average increases of 4.27%, 7.92%, 3.42%, and 8.01% in predictive accuracy for PH, FT, GTW, and GY, respectively. These results indicated that thoroughly considering trait‐specific genetic features (All‐TAMs) in predictive models could enhance the predictive accuracy of the model, with environment‐responsive genetic features (E‐TAMs) contributing to the pronounced improvement to predictive accuracy.

Alternatively, neglecting genetic feature categories and using both genetic feature and Raw_EPs as features for model training significantly improved predictive accuracy of each trait compared with no EPs (No_EPs). Furthermore, the inclusion of DR_EPs instead of Raw_EPs further enhanced in predictive accuracy (Figure 5a). Overall, using both All‐TAMs and DR_EPs as features resulted in the highest predictive accuracy of the model (Figure 5a). Compared with All‐SNPs, using both All‐TAMs and DR_EPs increased the predictive accuracy from 0.736 to 0.878 for PH (a 19.29% increase, percent change = (0.878–0.736)/0.736×100%), from 0.776 to 0.975 for FT (a 23.32% increase, percent change = (0.975–0.776)/0.776×100%), from 0.756 to 0.862 for GTW (a 14.02% improvement, percent change = (0.862–0.756)/0.756×100%), and from 0.482 to 0.619 for GY (a 28.42% increase, percent change = (0.619–0.482)/0.482×100%). Taken together, these results indicated that the use of All‐TAMs and RD_EPs as features could enhance the accuracy of predictive models, emphasizing the informative value of environmental data in genomic predictions and importance of feature processing in genetic and environmental data.

We then conducted SHAP analysis to evaluate the importance of features in models trained with All‐TAMs and RD_EPs. We selected the ten features that accounted for the greatest percent of phenotypic variation among traits for subsequent analysis. The results showed that the top 10 features contributing to FT phenotypic variation were all EPs. For GTW, seven of the top 10 features were EPs, while six of the top 10 were EPs for PH. The top 10 features responsible for variation in GY were evenly split between genetic and EPs (Figure 5b). Correspondingly, variance analysis indicated that E provided a strikingly higher contribution to variation in FT phenotype than G (88.0% vs 7.4%). E also accounted for a larger proportion of the phenotypic variation in GTW and PH than G, though to a less pronounced extent (≈43.0% vs 30.0%). By contrast, the contributions of E and G to phenotypic variation in GY were roughly equivalent (22.0% vs 18.7%, respectively). Notably, GY was more susceptible to multiple EPs (PAR, RH, PRE, GDD, and PTR) than other traits, which could explain its relatively low heritability. These SHAP findings aligned well with our analysis of trait variance and further uncovered the prominent influence of EPs in determining phenotype for some environment‐responsive maize traits, underscoring their potential for application in genomic selection breeding programs.

Among genetic features, the top four features contributing to PH were all Main‐TAMs; two of the top three features (S2_18656506 and S5_209487005) contributing to GW were Main‐TAMs; and three of the top five features (S3_184263156, S6_167214564, and S8_9279105) contributing to GY were its Main‐TAMs (Figure 5b). These results thus indicated that Main‐TAMs appear to contribute more to regulating traits than E‐TAMs.

In addition, comparison of absolute SHAP value rankings between the identified TAMs (including Main‐TAMs, G×E‐TAMs, and PP‐TAMs) and 2000 randomly selected SNPs from across the genome indicated that all TAM categories had significantly higher mean absolute SHAP scores than those of randomly selected SNPs (*P* < 0.05; Figure S9, Supporting Information). These findings were consistent with our GWAS results and provided further evidence that TAMs identified through our framework substantially contribute to both improving model performance and explaining phenotypic variation.

### 2.6 Genomic Selection Model Based on Automated Machine Learning Demonstrates Higher Predictive Accuracy than Other Current Models Across Diverse Prediction Tasks

**Figure 6:**
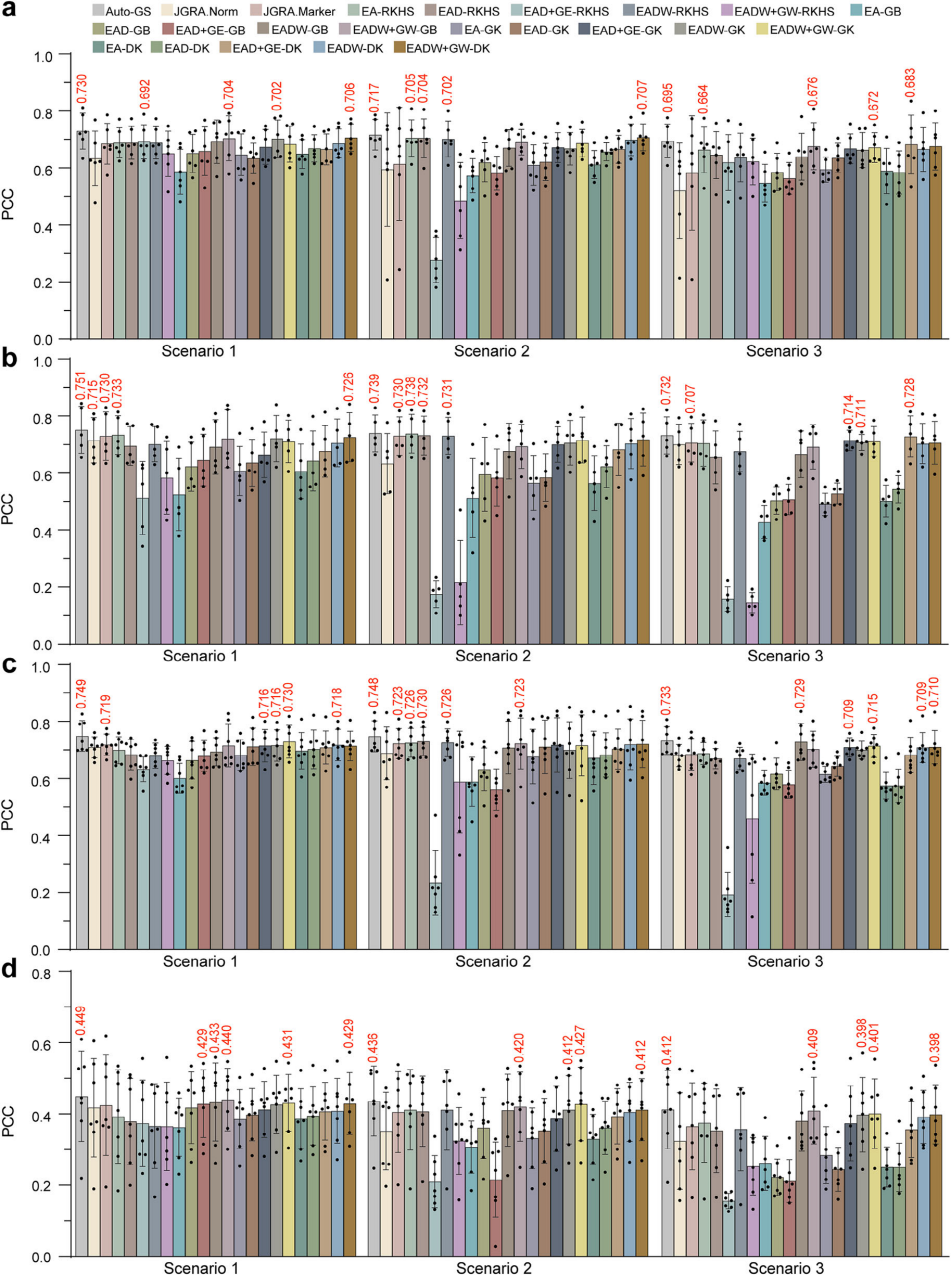
Comparative analysis of prediction accuracy between genomic selection model based on automated machine learning and other statistical models across diverse predictive scenario. Scenario 1: Phenotype prediction of untested genotypes in tested environments. Scenario 2: Phenotype prediction of tested genotypes in untested environments. Scenario 3: Phenotype prediction of untested genotypes in untested environments. Auto‐GS: Genomic selection model based on automated machine learning. JGRA.Norm: Joint genomic regression analysis using reaction‐norm parameters. JGRA.Marker: Joint genomic regression analysis using genome‐wide marker effect continua. EA‐GB, EA‐GK and EA‐DK: main additive‐effect model based on genomic best linear unbiased prediction (GBLUP), gaussian kernel and deep kernel. EAD‐GB, EAD‐GK and EAD‐DK: main additive plus dominance effects model based on GB, GK and DK. EAD+GE‐GB, EAD+GE‐GK and EAD+GE‐DK: main‐effect EAD plus G×E deviation model based on GB, GK and DK. EADW‐GB, EADW‐GK and EADW‐DK: main‐effect EAD with main envirotype information model based on GB, GK and DK. EADW+GW‐GB, EADW+GW‐GK and EADW+GW‐DK: main‐effect EADW plus reaction norm for GE model based on GB, GK and DK. a) Model performance comparison for plant height prediction in diverse scenarios. b) Model performance comparison for flowering time prediction in diverse scenarios. c) Model performance comparison for grain test weight prediction in diverse scenarios. d) Model performance comparison for grain yield prediction in diverse scenarios.

To compare our model with other current models in cross‐environment and cross‐genotype prediction tasks, we carried out phenotypic predictions for three distinct scenarios: Scenario 1) Phenotype prediction of untested genotypes in tested environments; Scenario 2) Phenotype prediction of tested genotypes in untested environments; and Scenario 3) Phenotype prediction of untested genotypes in untested environments. For these analyses, we tested 23 models, including genomic selection model based on automated machine learning (Auto‐GS); joint genomic regression analysis using reaction‐norm parameters (JGRA.Norm) or genome‐wide marker effect continua (JGRA.Marker); main additive‐effect model based on reproducing kernel hilbert space, genomic best linear unbiased prediction (GBLUP), gaussian kernel and deep kernel (EA‐RKHS, EA‐GB, EA‐GK and EA‐DK), main additive plus dominance effects model based on RKHS, GB, GK and DK (EAD‐RKHS, EAD‐GB, EAD‐GK and EAD‐DK), main‐effect EAD plus G×E deviation model based on RKHS, GB, GK and DK (EAD+GE‐RKHS, EAD+GE‐GB, EAD+GE‐GK and EAD+GE‐DK), main‐effect EAD with main envirotype information model based on RKHS, GB, GK, and DK (EADW‐RKHS, EADW‐GB, EADW‐GK and EADW‐DK), and main‐effect EADW plus reaction norm for GE model based on RKHS, GB, GK, and DK (EADW+GW‐RKHS, EADW+GW‐GB, EADW+GW‐GK, and EADW+GW‐DK). The findings revealed that our Auto‐GS model consistently achieved the highest predictive accuracy across all traits, irrespective of predictive scenario (**Figure** **6**).

In prediction Scenario 1, Auto‐GS model demonstrated the highest average accuracy in FT predictions across environments (PCC = 0.751), followed by GTW (PCC = 0.749), PH (PCC = 0.730), and GY (PCC = 0.449). The accuracy of FT prediction was 2.88% higher than that of the second‐ranked model EA‐RKHS. The GTW prediction accuracy was 2.60% higher than that of the second‐ranked model, EADW+GW‐GK. Additionally, the PH prediction accuracy was 3.40% higher than the second‐ranked model, EADW+GW‐DK. The GY prediction accuracy by Auto‐GS model also surpassed the second‐ranked model, EADW+GW‐GB, by 2.05%. These results indicated that Auto‐GS model showed strong performance in cross‐genotype predictions (Figure 6). In prediction Scenario 2, Auto‐GS model again showed the highest average accuracy in GTW predictions (PCC = 0.748), followed by FT (PCC = 0.739), PH (PCC = 0.717), and GY (PCC = 0.436). Its predictive accuracy in GTW was 2.47% higher than that of the second‐ranked model EAD‐RKHS. The FT prediction accuracy was 0.14% higher than that of the second‐ranked model, EA‐RKHS. Additionally, the PH prediction accuracy was 1.41% higher than the second‐ranked model, EADW+GW‐DK. Auto‐GS model also showed higher accuracy in predicting GY than the next best model, EADW+GW‐GK, by 2.11%. These results demonstrated Auto‐GS model's robust performance in cross‐environment predictions (Figure 6). In prediction Scenario 3, Auto‐GS model again showed the highest average predictive accuracy for GTW (PCC = 0.733), followed by FT (PCC = 0.732), PH (PCC = 0.695), and GY (PCC = 0.412). The accuracy of GTW prediction was 0.55% higher than that of the second‐ranked model, EADW‐GB. The FT prediction accuracy was 0.55% higher than that of the second‐ranked model, EAD+GE‐DK. Additionally, PH prediction accuracy was 1.76% higher than the second‐ranked model, EAD+GE‐DK, and predictions of GY had 0.73% higher accuracy than the next most accurate model, EADW+GW‐GB. These results indicated that Auto‐GS model displayed consistent performance in cross‐genotype and cross‐environment prediction tasks (Figure 6). These results indicated that Auto‐GS model could handle a variety of complex predictive tasks with higher accuracy than any other current models, highlighting its immense potential in crop genomic prediction breeding.

We subsequently compared the computational efficiency of our Auto‐GS model with that of 22 alternative models across three prediction scenarios. This analysis revealed that computational time consistently increases with expanding dataset size across all models (e.g., FT contains data from 5 environments, 6 environments for PH, and 7 environments each for GY and GTW). Models based on RKHS, GB, GK, and DK frameworks required significantly longer computation time compared to JGRA.norm, JGRA.Marker, and Auto‐GS (Figure S10, Supporting Information). Among them, JGRA.norm achieved the shortest computation time across all traits and prediction scenarios, followed by JGRA.Marker, and Auto‐GS ranking third in computational efficiency. Notably, the EAD+GE‐RKHS model exhibited the longest computation duration in Scenario 1 and 3, the EADW+GW‐GB model exhibited the longest computation duration in Scenario 2. For instance, JGRA.norm completed prediction Scenario 1 for FT traits in 89 s, while JGRA.Marker required 146 s, and Auto‐GS took 440 s. In contrast, EAD+GE‐RKHS required 129348 s for this scenario. Similarly, the computation times recorded for prediction Scenario 1 for GY were 89 s (JGRA.norm), 178 s (JGRA.Marker), 656 s (Auto‐GS), and 168147 s (EAD+GE‐RKHS). These results indicate that Auto‐GS provides substantially enhanced computational efficiency in genomic prediction scenarios compared to conventional genomic prediction approaches.

### 2.7 Genomic Selection Model Based on Automated Machine Learning Exhibits Good Scalability in Predicting Phenotypes Across New Datasets

To test the scalability of Auto‐GS model in predicting phenotypes across new datasets, we used Maize180, Maize331, and Maize359 as independent test sets to carry out cross‐genotype and cross‐environment prediction, in which Maize180 represented untested genotypes in tested environments; Maize331 represented tested genotypes in untested environments; and Maize359 represented untested genotypes in untested environments (see Tables S2 and S8–S10, Supporting Information, for a detailed list of their respective EPs and trait values).

**Figure 7:**
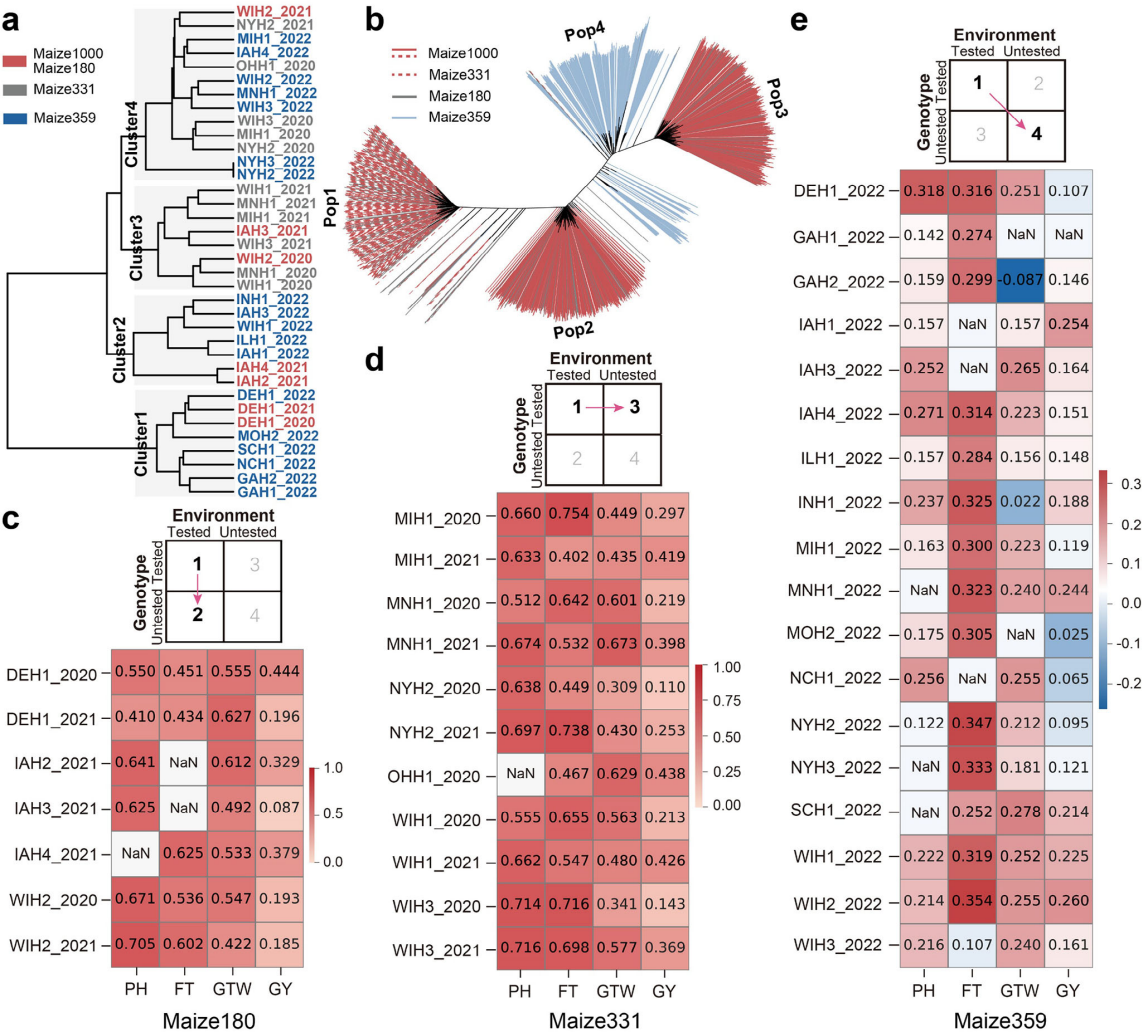
Multi‐environment joint genomic prediction cross‐environments and cross‐genotypes. a) Environmental clustering tree for all 36 cultivated environments. Different font colors represent cultivated environments from different datasets. b) Neighbor‐joining tree for all 1539 maize hybrids. Different line styles and colors represent genotypes from different datasets. c) Multi‐environment joint phenotypic prediction cross‐genotypes. d) Multi‐environment joint phenotypic prediction cross‐environments. e) Multi‐environment joint genomic prediction cross‐environments and cross‐genotypes. NaN indicates missing Pearson's correlation coefficient due to a lack of observed phenotypic data. PH: plant height; FT: flowering time; GTW: grain test weight; GY: grain yield.

Clustering analysis showed that all 36 environments could be grouped into four clusters. Maize1000 and Maize180 environments were distributed across the four clusters, while Maize331 environments were primarily distributed in Clusters 3 and 4, and the Maize359 environments were mainly distributed in Clusters 1, 2, and 4 (**Figure** **7**a). At the same time, Neighbor‐joining (NJ) tree revealed that all genotypes could be classified into four groups. Maize1000 and Maize180 included individuals from Pop1, Pop2, and Pop3, while Maize331, as a subset of Maize1000, was distributed within Pop1. By contrast, Maize359 was distributed in Pop4 and showed a relatively distant genetic relationship with Maize1000, Maize180, and Maize331 (Figure 7b). These analyses revealed similarity in the relationships among environments and among genetics across different test sets.

We then trained an optimized model integrating All‐TAMs and DR_EPs of Maize1000 for independent prediction. In the Maize180 test set, the PCC values for PH ranged from 0.410 to 0.705 across environments, from 0.451 to 0.625 for FT predictions, and from 0.422 to 0.627 in GTW predictions. However, predictions of GY had lower PCC values and showed markedly greater variation across various environments, ranging from 0.087 to 0.444 (Figure 7c). Similarly, in the Maize331 test set, the PCC values reached 0.555–0.716, 0.402–0.750, 0.309–0.673, and 0.110–0.438, for PH, FT, GTW, and GY across different environments, respectively (Figure 7d). As expected, the Maize359 dataset resulted in poorer prediction outcomes in which none of the PCC values exceeded 0.400 for any trait across all environments, and the PCC values for GTW and GY did not exceed 0.300 (Figure 7e). It is likely that the distant genetic relationship between Maize359 and the training population, Maize1000, was primarily responsible for this outcome. In addition, we further validated the generalizability of our model using the previously published large scale Maize2808 hybrid dataset, including the Maize366 and Maize5844 datasets, and a Wheat286 dataset (see detailed analyses in Supporting Results File, Tables S11–S18 and Figures S12–S14, Supporting Information). This analysis demonstrated that applying environmental data to incorporate PP and G×E interactions in genomic selection and genetic analysis with our Auto‐GS model could further improve phenotypic predictions in large‐scale hybrid screening. In summary, these findings underscore that Auto‐GS model shows good scalability in predicting phenotypes across new datasets, but its effectiveness requires considerable environmental and genetic similarity between the training set and independent test sets.

## 3 Discussion

Environment plays a pivotal role in influencing phenotypic variation. Our variance analysis in a multi‐environment trial data from 1000 maize hybrids revealed that environment and G×E interactions have significantly greater influence on variation in PH, FT, GTW, and GY than that of genotype. In particular, phenotypic variance in FT is predominantly driven by environmental influences, whereas variance in GY is largely governed by G×E interactions (Figure 1d). These results align well with prior research,^[^ ^16^, ^18^ ^]^ suggesting that this phenomenon is universal across diverse maize hybrid populations. These findings, together with previous studies, collectively underscore the prominent role of environment in shaping maize phenotype, thus emphasizing the informative value of environmental data in genetic analysis and breeding of maize.

To integrate environmental data into genetic analysis of loci controlling PP, Li et al. (2018) proposed a method that establishes a reaction norm between EPs and phenotypes to characterize the impact of a given EP on PP within a certain time window,^[^ ^17^ ^]^ where plants in different environments are at different developmental stages. Despite its wide application, this method shows limited ability to explain the influence of EPs on PP in specific stages of plant development.^[^ ^18^, ^19^, ^20^, ^21^, ^22^ ^]^ To address this challenge, our strategy in the current study establishes a reaction norm between EPs and phenotypes by first reducing the dimensionality of EPs in different environments to 36 developmental stages of maize (i.e., V0‐R6, Figure 3; Figure S1, Supporting Information), then establishes a reaction norm between EPs and phenotype based on these RD_EPs. The resulting reaction norm can therefore reflect the impact of EPs on PP during a given developmental stage, as all plants are at the same developmental stage across different environmental conditions (Figure 4b). This capacity to predict the contribution of PP‐related loci to variation in a given trait at a specific developmental stage can thus provide a valuable supplement to the strategy developed by Li and colleagues (2018).^[^ ^17^ ^]^

In previous studies, single EPs most strongly correlated with traits have been typically used to calculate PP parameters for GWAS or QTL mapping.^[^ ^18^, ^19^, ^20^, ^21^, ^22^ ^]^ However, results in this current study indicate that employing PP parameters derived from multiple EPs in GWAS can uncover a greater number of PP‐TAMs than any single EP (Figure 4c; Table S5, Supporting Information). Although a considerable proportion of TAMs detected by different EPs overlap, many TAMs are unique to an individual EP, and the reliability of such EP‐specific TAMs was supported by their association with known functional genes (Table S5, Supporting Information). Moreover, the identification of such unique TAMs illustrates the advantage of genetic analysis with PP parameters derived from multiple EPs. In addition, multi‐environment joint GWAS to identify TAMs controlling G×E interactions in our study showed that very few loci overlap between PP and G×E, which is consistent with reports in other species,^[^ ^48^, ^49^ ^]^ and suggests that PP and G×E interactions likely have different genetic basis. Overall, these results emphasize the need to adopt different approaches to mine environment‐responsive genetic loci affect maize phenotype.

Incorporating environmental data into genomic prediction is now widely accepted to enhance accuracy in predictive modeling.^[^ ^29^, ^30^, ^31^, ^50^, ^51^ ^]^ In addition, numerous studies have used TAMs as features in genomic prediction.^[^ ^26^, ^27^, ^28^ ^]^ Analysis of our genomic predictions confirmed conclusions of previous studies, and further demonstrated that incorporating EPs with reduced dimensionality based on maize developmental stages could significantly improve predictive accuracy (Figure 5), potentially due to the close relationship maize growth patterns with developmental stage that renders these EPs informative of likely phenotype. Additionally, we found that using Main‐TAMs as features yields similar predictive accuracy to E‐TAMs, whereas using both Main‐TAMs and E‐TAMs together obviously increases predictive accuracy, even surpassing models that use All‐SNPs as features (Figure 5). These results highlight the value of E‐TAMs in genomic prediction and support the application of E‐TAMs in breeding.

As ML models have shown strong capacity to process large‐scale datasets, synthesize multi‐omics data, and capture complex feature relationships,^[^ ^52^, ^53^, ^54^ ^]^ we employed an AutoML framework to construct a multi‐environment joint predictive model for PH, FT, GTW, and GY traits, which enabled the inclusion of all RD_EPs in the predictive model, providing rich environmental feature information. In contrast, other models, such as that of Li et al. (2021),^[^ ^21^ ^]^ only permit the use of a single EP most correlated with phenotype in a specific time window to build predictive models. Moreover, AutoML framework also permits the selection of various base machine learning algorithms for model training and ensembling, thereby enhancing the predictive performance of the model. In various complex prediction tasks, our model consistently outperformed seven statistical prediction methods across different traits and prediction tasks (Figure 6), thus demonstrating its strong potential for applying in crop breeding. Moreover, predictions based on three independent test datasets indicated that our Auto‐GS model also exhibits good predictive ability with new datasets and shows excellent scalability (Figure 7). However, it should be noted that prediction accuracy still largely depends on genetic and environmental relationships between the training and test sets, which is a challenge commonly faced by all current genomic prediction methods.^[^ ^55^, ^56^ ^]^ Solutions to this issue may lie in the design of breeding experiments.^[^ ^57^, ^58^, ^59^ ^]^ In summary, our research highlights the advantages of Auto‐GS model over statistical models in genomic prediction.

The theoretical basis for the superiority of our genomic selection model over conventional models partially lies in the integration of RD_EPs. The reduction of EPs to RD_EPs aligned with specific developmental stages allows the model to better capture the nuanced effects of EPs across different growth phases. Conventional models often face the challenge to incorporate the environment in a dynamic manner, instead using static or averaged environmental data that do not account for temporal changes in plant‐environment interactions. By contrast, our approach dynamically aligns environmental conditions with maize developmental stages, providing a more precise understanding of how these conditions impact traits like FT and GY. Furthermore, the dimensionality reduction of EPs was carefully designed to ensure that the reduced parameters are biologically meaningful, as they correspond to key developmental stages in maize growth. By aligning EPs with these stages, we reduce noise and focus on the most relevant EPs that influence phenotypic variation. This dimensionality reduction also helps mitigate overfitting, a common issue in high‐dimensional data analysis, leading to more stable and generalizable predictions.

While this study focuses on maize, the methods employed here are broadly applicable to other crops. The concept of integrating environmental data into genomic selection models can be adapted to crops like wheat, rice, and soybean, which also experience significant G×E interactions. The use of AutoML in genomic prediction models provides a scalable and flexible framework that can be customized to other species by adjusting developmental stages and EPs specific to each crop. By adopting this approach, breeding programs worldwide could be able to accelerate the development of climate‐adaptive varieties, enhancing food security in the face of global environmental change.

The application of environment‐responsive genetic loci in crop breeding is a key strategy for improving adaptability. However, prior to introgression, such loci require validation of their biological relevance and identification of their causal genes. Here, we identified numerous G×E‐TAMs and PP‐TAMs, and conducted preliminary validation of their contributions to phenotypic variation through extensive statistical analyses and SHAP interpretation. Although the results supported the reliability of these loci, the genes underlying their respective effects have yet to be identified. Future work can address this knowledge gap through systematic functional characterization of these loci. For example multi‐environment transcriptomic analyses can be used to prioritize candidate genes associated with G×E‐TAMs and PP‐TAMs. Maize mutant libraries and/or targeted gene‐editing can be employed in multi‐environment phenotypic evaluations to verify gene functions at scale. This functional validation of environment‐responsive genes can facilitate their subsequent deployment in breeding programs aimed at developing climate‐resilient maize hybrids.

## 4 Experimental Section

### Germplasm Accessions and Their Phenotypes

Dataset I: The 1539 maize hybrids derived from the G2F dataset,^[^ ^41^, ^42^ ^]^ which were divided into four subsets: Maize1000, Maize180, Maize331, and Maize359. Maize1000 comprised 1000 hybrids phenotyped across seven environments in 2020 and 2021, serving as the training population for GWAS and genomic selection. Maize180, consisting of 180 hybrids phenotypically assessed in the same environments, was employed to test model performance and predict phenotypes across genotypes. This dataset is referred to as the untested genotypes in tested environments. Maize331, a subset of 331 hybrids from the Maize1000 dataset, underwent phenotypic evaluation in 11 additional environments in 2020 and 2021. This dataset was used to test model performance and conduct predictions across environments referred to as the tested genotypes in untested environments. Maize359 included 359 hybrids phenotypically evaluated in 18 additional environments in 2022. This dataset was used to test model performance and conduct genomic prediction across environments and genotypes, and was referred to as the untested genotypes in untested environments. Detailed descriptions of the phenotypic experimental conditions are detailed in Table S1 (Supporting Information). Tested phenotypes include PH, FT, GTW, and GY.

Dataset II: The 8652 maize hybrids were derived from 30 F_1_ populations, which were generated by crossing 1428 inbred lines from the CUBIC (Complete‐diallel plus Unbalanced Breeding‐derived Inter‐Cross) synthetic population as a maternal pool with 30 paternal testers from diverse heterotic groups.^[^ ^60^ ^]^ Phenotypic evaluation of PH, FT, and ear weight (EW) traits was conducted in the 8652 hybrids across five locations in Northern China (Yushu, Jilin Province, 43°42′N, 125°18′E; Shenyang, Liaoning Province, 42°03′N, 123°33′E; Beijing, 40°10′N, 116°21′E; Baoding, Hebei Province, 38°39′N, 115°51′E, and Xinxiang, Henan Province, 35°27′N, 114°01′E) over two growing seasons (2014 and 2015). The hybrids were partitioned into three datasets: Maize2808, Maize366, and Maize5844. Maize2808 underwent phenotypic evaluation in 2014, Maize366, a subset of Maize2808, was evaluated in 2015, and Maize5844, which has no genotypic overlap with the other two datasets, was also evaluated in 2015. Maize2808 was used for GWAS analysis and construction of the genomic prediction model, Maize366 was used to test cross‐environment predictive ability, and cross‐genotype and cross‐environment predictive capabilities were assessed using Maize5844.

Dataset III: The wheat dataset included 286 high‐yielding, advanced elite lines of spring wheat (Wheat286), with PH, FT, and yield (YLD) evaluated over four growing seasons (from 2009 to 2013) at the CIMMYT experimental station near Ciudad Obregón, Sonora State, in Northwest Mexico (27.20°N, 109.54°W).^[^ ^61^ ^]^ This dataset served as the training population for GWAS and genomic selection.

### Heritability of Traits

The broad‐sense heritability (*H* ^2^) of PH, FT, GTW, and GY in the Maize1000 dataset was calculated using the formula: $H^{2}=\sigma_{g}^{2}\;/\left(\sigma_{g}^{2}+\frac{\sigma_{e}^{2}}{L}\right)$, with the function lmer(Trait∼(1|Line)+(1|Env)) in the R package “lme4”.^[^ ^62^ ^]^ Here, $\sigma_{g}^{2}$ represents genetic variance, $\sigma_{e}^{2}$ represents environmental variance, and L represents the number of environments.

### Environmental Data

Environmental data were downloaded from the NOAA (https://www.worldclim.org/) and WorldClim (https://power.larc.nasa.gov) databases based on the location and dates of each experiment. These data include DL, daily maximum and minimum temperatures, RH, PRE, and PAR. GDD from the date of sowing were calculated using the formula: *GDD*  = (*T*_max_ + *T*_min_) ÷ 2 − *T*_base_, where *T*_max_​ is the daily maximum temperature (86°F), *T*_min_ is the daily minimum temperature, and *T*_base_​ is the base temperature for activity (50°F). If the *T*_max_ exceeds 86°F, it is set to 86°F, and if the *T*_min_ exceeds the *T*_base _, it is set to 50°F. Four derived parameters—DTR, PTS, PTR, and PTT—were calculated using the following formulas: *DTR*  = *T*_max_  − *T*_min_; $PTS=\left(T_{\max}^{2}-T_{\min}^{2}\right)\;\times DL^{2}$; *PTR*  =  *GDD* ÷ *DL*; *PTT*  =  *GDD* × *DL*.

### Automated Machine Learning Framework

An AutoML framework was designed to integrate data processing, environmental feature handling, GWAS, model training, and phenotypic prediction. The data processing and feature handling functions performed genotype quality control, dimensionality reduction of EPs, and calculation of PP parameters. GWAS was conducted using the 3VmrMLM method^[^ ^43^ ^]^ to identify TAMs associated with specific traits, which were then used as genetic features in model training along with RD_EPs. To train the model, a variety of base machine learning models were integrated, using the Optuna automated hyperparameter tuning algorithm to optimize the model parameters, a stacking algorithm for model ensembling to improve predictive accuracy, and SHAP to enhance model interpretability.^[^ ^44^, ^45^, ^46^ ^]^ The phenotype prediction function facilitated validation using independent test data, including predictions for untested genotypes in a tested environment, tested genotypes in untested environments, and untested genotypes in untested environments. PCC was used to evaluate the predictive accuracy of the final model.

### Genotype Data Processing

The raw genotype data of Dataset I included 437214 SNPs. First, the Maize1000 dataset was filtered and used to inform the filtering of the other three maize subsets. SNPs with a missing rate greater than 30% were removed from Maize1000. Missing genotypes were imputed using Beagle software.^[^ ^63^ ^]^ Multiallelic SNPs and those with genotype frequencies (AA, Aa, and aa) below 0.02 at each locus were excluded. After filtering, 149719 high‐quality SNP markers were retained. This retained set of SNPs was then applied as an index for the Maize180, Maize331, and Maize359 datasets to filter other SNPs, ensuring all retained SNPs matched those in Maize1000 dataset. Finally, all datasets retained 149719 SNPs for subsequent analysis.

The raw genotype data of Dataset II comprised 4549828 high‐quality, imputed SNPs. Due to the excessively large number of markers, which hindered efficient analysis, we followed the report by Yang et al. (2022)^[^ ^64^ ^]^ and extracted 156269 high‐quality SNPs for subsequent analysis. At the same time, genotyping in Dataset III was performed by Illumina iSelect 90K SNP assay, yielding data for 26814 SNPs.^[^ ^61^ ^]^

### Dimensionality Reduction of Environmental Data

The dimensionality reduction process for environmental data involved two main steps. First, the growth period of each genotype was divided into 36 development stage‐environment windows (36 windows for maize and 11 for wheat) based on the relationship between the accumulated GDD and the development stage.^[^ ^47^, ^65^ ^]^ Then, the development stage‐environment windows of each genotype were used to segment other EPs such as DL, RH, PRE, PAR, DTR, PTS, PTR, and PTT. Finally, the average environmental data for each EP were then calculated within each development stage‐environment window, resulting in a single value per EP per window.

### Calculate Phenotypic Plasticity Parameters

The environmental mean for each trait was calculated in every environment. The mean of each EP was calculated using a sliding window approach, where windows spanned point A to point B, which represents the development stage‐environment windows. The Pearson's correlation coefficient between each trait's environmental mean and mean EP of point A to point B was calculated. Windows with the highest correlations (*r* ≥ 0.90 or *r* ≤ −0.90) were identified and used for linear regression analysis of the phenotypic values of each genotype across all environments. The resulting linear equation provided the intercept and slope, defined as two PP parameters, for each genotype and used in subsequent GWAS.

### Genome‐Wide Association Analysis

GWAS was conducted on the Maize1000, Maize2808, and Wheat286 dataset using the 3VmrMLM method, which incorporates the first three principal components and the K matrix to control for false positives.^[^ ^43^ ^]^ This method conducts both single‐ and multi‐environment GWAS, detecting main effects and environment interaction TAMs. It also estimates the additive and dominance effects of TAMs, along with their environmental interactions. For this study, multi‐environment GWAS was carried out using multi‐environment phenotypes for each trait to identify the main effect and environment interaction TAMs controlling these traits. Additionally, single‐environment GWAS was conducted using PP parameters (intercept and slope) for the four traits to detect TAMs influencing PP.

### Genomic Prediction

An ensemble genomic prediction model was generated with a focus on regression tasks, utilizing four base models known for their efficacy in tabular data prediction: CatBoost, XGBoost, LightGBM, and BayesianRidge.^[^ ^66^, ^67^, ^68^, ^69^ ^]^ During model training, hyperparameters were optimized using the Optuna and Tree‐Structured Parzen Estimator (TPE) optimization algorithms,^[^ ^44^ ^]^ a stacking algorithm for model ensembling to improve predictive accuracy of the final model.^[^ ^45^ ^]^ SHAP interpretability technology was employed to interpret and provide biological explanations for the model features.^[^ ^46^ ^]^ Using the Maize1000, Maize2808, and Wheat286 dataset for training, detected TAMs were divided into two categories: Main‐TAMs and E‐TAMs (including G×E‐TAMs and PP‐TAMs). SNP markers identified within the upstream and downstream regions of the TAMs for each trait, using intervals defined by either an LD threshold of *r* ^2^≥0.5 or half the maximum LD decay distance. These SNP markers, along with the calculated RD_EP values (nine EPs across 36 windows for maize hybrid and nine EPs across 11 windows for wheat), were applied as features for constructing a genomic prediction model. Five‐fold cross‐validation was conducted for all analyses, with Pearson's correlation coefficient between observed and predicted values employed to assess the model's predictive performance. The constructed All‐TAMs plus RD_EPs model was subsequently tested with the different datasets (Maize180, Maize331, Maize359, Maize366, and Maize5844) to evaluate performance in multi‐environment joint prediction across genotypes and environments.

### Population Structure Analysis

To clarify genetic relationships, phylogenetic trees were constructed for the Maize1000, Maize180, and Maize359 datasets using the Neighbor‐Joining function in Tassel.^[^ ^70^, ^71^ ^]^ The trees were visualized and refined using the iTOL online tool (https://itol.embl.de/). Linkage disequilibrium analysis of the Maize1000 dataset was conducted with PopLDdecay, using all default parameters except for the MaxDist, which was adjusted to 5000.^[^ ^72^ ^]^ Principal component analysis (PCA) of the Maize1000 dataset was performed using Plink software to elucidate its population genetic structure.^[^ ^73^ ^]^

### Environmental Clustering Analysis

All genotype datasets (Maize1000, Maize180, Maize331, and Maize359) were phenotypically assessed across 36 environments. Environmental clustering analysis was performed using the parameter values of nine EPs across these 36 development stage‐environment windows. The Euclidean distance algorithm^[^ ^74^ ^]^ was employed to calculate the relationships between environments, and Ward's method^[^ ^75^ ^]^ was used for environmental clustering.

### Model Comparison Methods

The prediction accuracy of Auto‐GS model was compared with that of the JGRA (including JGRA.Norm and JGRA.Marker) model, main additive‐effect model based on reproducing kernel hilbert space, genomic best linear unbiased prediction, gaussian kernel and deep kernel (EA‐RKHS, EA‐GB, EA‐GK and EA‐DK), main additive plus dominance effects model based on RKHS, GB, GK and DK (EAD‐RKHS, EAD‐GB, EAD‐GK and EAD‐DK), main‐effect EAD plus G×E deviation model based on RKHS, GB, GK and DK (EAD+GE‐RKHS, EAD+GE‐GB, EAD+GE‐GK and EAD+GE‐DK), main‐effect EAD with main envirotype information model based on RKHS, GB, GK and DK (EADW‐RKHS, EADW‐GB, EADW‐GK and EADW‐DK), and main‐effect EADW plus reaction norm for GE model based on RKHS, GB, GK and DK (EADW+GW‐RKHS, EADW+GW‐GB, EADW+GW‐GK and EADW+GW‐DK). These existing models efficiently leverage environmental data for genomic prediction.^[^ ^18^, ^19^, ^20^, ^21^, ^22^, ^51^, ^76^ ^]^ The predictive accuracy of the models was assessed as follows: 1) untested genotypes in the tested environment were compared using leave‐one‐half‐of‐genotypes‐out cross‐validation; 2) tested genotypes in untested environments were compared using leave‐one‐environment‐out cross‐validation; and 3) untested genotypes in untested environments were compared using joint leave‐one‐environment‐out and one‐half‐of‐genotypes‐out cross‐validation.^[^ ^17^, ^19^, ^22^ ^]^ Prediction accuracy was calculated as the PCC between observed and predicted values.

## Conflict of Interest

The authors declare no conflict of interest.

## Author Contributions

K.H. and T.Y. contributed equally to this work. H.L. initiated the project and designed the study. K.H., T.Y., and S.C. performed data processing. K.H. and T.Y. performed genetic and genomic selection analysis. K.H., T. Y., and H.L. drafted the manuscript. S.G., L.L., X.Z., C.H., Y.X., J.W., P.B.M., H.S., and L.X. provided valuable advice for the experimental design, results interpretation and manuscript modification. K.H., T.Y., S.G., and H.L revised the manuscript. All authors read and approved the manuscript.

## Supporting information



Supporting Information

Supplemental Tables

## Data Availability

The data supporting the findings of this study are available within the paper itself or provided in the Supplementary Materials. Phenotypic and genotypic data for maize hybrids are available on The Genomes To Fields Initiative download page (https://www.genomes2fields.org/resources/) and link https://ftp.cngb.org/pub/CNSA/data3/CNP0001565/zeamap/99_MaizegoResources/01_CUBIC_related/. Phenotypic and genotypic data for wheat can be downloaded from the link http://hdl.handle.net/11529/10714. Scripts used in this study are available at GitHub (https://github.com/AIBreeding/AutoGS).
