# Supplementary material for: Leveraging Automated Machine Learning for Environmental Data‐Driven Genetic Analysis and Genomic Prediction in Maize Hybrids
Source: Adv Sci (Weinh). 2025 Mar 6;12(17):2412423. doi: 10.1002/advs.202412423 (PMC12061318; doi:10.1002/advs.202412423)
Supplement: Supplementary file 1 — Supporting Information [file ADVS-12-2412423-s001.docx]

**Leveraging Automated Machine Learning for Environmental Dimension-Data Genetic Analysis and Genomic Prediction in Maize Hybrids**

Kunhui He^1,2,^^†^, Tingxi Yu^1,2,†^, Shang Gao^1,2^, Shoukun Chen^1,2^, Liang Li^1^, Xuecai Zhang^1,2,3^, Changling Huang^1,2^, Yunbi Xu^1^, Jiankang Wang^1,2^, Boddupalli M. Prasanna^4^, Sarah Hearne^3^, Xinhai Li^1^, Huihui Li^1,2*^

^1^ State Key Laboratory of Crop Gene Resources and Breeding, Institute of Crop Sciences, Chinese Academy of Agricultural Sciences (CAAS), CIMMYT-China office, Beijing, 100081, China.

^2^ Nanfan Research Institute, CAAS, Sanya, Hainan, 572024, China.

^3^ International Maize and Wheat Improvement Center (CIMMYT), Apdo. Postal 6-641, Texcoco, D.F. 06600, Mexico

^4^ CIMMYT, International Centre for Research in Agroforestry (ICRAF) House, Nairobi 00100, Kenya

^†^ These authors contributed equally to this article.

^*^ Corresponding author: Huihui Li, email: lihuihui@caas.cn.

**Figure S1.** The relationship between maize hybrids development stages and growing degree days (GDD).

**Figure S2.** The line chart displays the trends in air relative humidity (RH), photothermal time (PTT), photothermal rate (PTR), daily diurnal temperature range (DTR), and photothermal sensitivity (PTS) across 36 development stage-environment windows.

**Figure S3.** Determination of critical development stage-environment windows for each trait and environmental parameter in the Maize1000 dataset. **a** Heatmaps of the correlations between four dimensionality-reduced environmental parameters (RD_EPs) and the population’s environmental mean of plant height (PH), flowering time (FT), grain test weight (GTW), and grain yield (GY). The asterisk on the heatmaps represents the selected critical window. **b** Linear regression of four RD_EPs (critical development stage-environment windows) with the population’s environmental mean values of PH, FT, GTW, and GY. **c** The critical development stage-environment windows for PH, FT, GTW, and GY. Highlight in red font for correlation coefficients greater than 0.90 or less than -0.90. DTR: daily diurnal temperature range; PAR: photosynthetically active radiation; PTR: photothermal rate; DL: day length; RH: air relative humidity; PRE: precipitation; GDD: growing degree days; PTS: photothermal sensitivity; PTT: photothermal time.

**Figure S4.** Heatmaps of the correlations between RD_EPs and the population’s environmental mean of plant height (PH), flowering time (FT), grain test weight (GTW), and grain yield (GY). DTR: daily diurnal temperature range; PAR: photosynthetically active radiation; PTR: photothermal rate; DL: day length; RH: air relative humidity; PRE: precipitation; GDD: growing degree days; PTS: photothermal sensitivity; PTT: photothermal time.

**Figure S5.** Linear regression of dimensionality-reduced environmental parameters (critical development stage-environment windows) with the population’s environmental mean values of plant height (PH), flowering time (FT), grain test weight (GTW), and grain yield (GY). DTR: daily diurnal temperature range; PAR: photosynthetically active radiation; PTR: photothermal rate; DL: day length; PRE: precipitation; GDD: growing degree days; PTS: photothermal sensitivity.

**Figure S6.** Phenotypic distributions of multi-environment phenotypic and phenotypic plasticity parameters for four traits. **a** Phenotypic distributions of multi-environment phenotypic for plant height (PH), flowering time (FT), grain test weight (GTW), and grain yield (GY). **b** The distributions of phenotypic plasticity parameters and linear regression between slop and intercept for PH, FT, GTW, and GY. DTR: daily diurnal temperature range; PAR: photosynthetically active radiation; PTR: photothermal rate; DL: day length; PRE: precipitation; GDD: growing degree days; PTS: photothermal sensitivity.

**Figure S7.** Multi-environment joint genome-wide association study for four traits. Main represents environmental stability trait-associated markers, G×E represents genotype-environment interactions trait-associated markers. The different colors of the dots in the plot represent different traits. PH: plant height; FT: flowering time; GTW: grain test weight; GY: grain yield.

**Figure S8.** Prediction accuracy of ensemble models with different model combinations for four traits. **a** Prediction performance of ensemble models composed of different models on plant height. **b** Prediction performance of ensemble models composed of different models on flowering time. **c** Prediction performance of ensemble models composed of different models on grain test weight. **d** Prediction performance of ensemble models composed of different models on grain yield. BR: BayesianRidge; LGB: Light Gradient Boosting Machine (LightGBM); CAT: ategorical Boosting (CatBoost); XGB: Extreme Gradient Boosting (XGBoost).

**Figure S9.** Quantifying the influence of genetic features on prediction accuracy using SHapley Additive exPlanations (SHAP) interpretation. **a** Mean absolute SHAP values of different genetic features in the plant height prediction model. **b** Mean absolute SHAP values of different genetic features in the flowering time prediction model. **c** Mean absolute SHAP values of different genetic features in the grain test weight prediction model. **d** Mean absolute SHAP values of different genetic features in the grain yield prediction model. Main-TAMs: environmental stability trait-associated markers. G×E-TAMs: genotype-environment interactions trait-associated markers. PP-TAMs: phenotypic plasticity trait-associated markers. SNPs: single nucleotide polymorphisms.

**Figure S10.** Comparative analysis of computational efficiency between genomic selection model based on automated machine learning and other statistical models across diverse predictive scenario. Scenario 1: Phenotype prediction of untested genotypes in tested environments. Scenario 2: Phenotype prediction of tested genotypes in untested environments. Scenario 3: Phenotype prediction of untested genotypes in untested environments. Auto-GS: Genomic selection model based on automated machine learning. JGRA.Norm: Joint genomic regression analysis using reaction-norm parameters. JGRA.Marker: Joint genomic regression analysis using genome-wide marker effect continua. EA-GB, EA-GK and EA-DK: main additive-effect model based on genomic best linear unbiased prediction (GBLUP), gaussian kernel and deep kernel. EAD-GB, EAD-GK and EAD-DK: main additive plus dominance effects model based on GB, GK and DK. EAD+GE-GB, EAD+GE-GK and EAD+GE-DK: main-effect EAD plus G×E deviation model based on GB, GK and DK. EADW-GB, EADW-GK and EADW-DK: main-effect EAD with main envirotype information model based on GB, GK and DK. EADW+GW-GB, EADW+GW-GK and EADW+GW-DK: main-effect EADW plus reaction norm for GE model based on GB, GK and DK. In a bar chart, each data point represents an experimental environment. **a** Comparison of computational efficiency among models for plant height prediction in diverse scenarios. **b** Comparison of computational efficiency among models for flowering time prediction in diverse scenarios. **c** Comparison of computational efficiency among models for grain test weight prediction in diverse scenarios. **d** Comparison of computational efficiency among models for grain yield prediction in diverse scenarios.

**Figure** **S11.** The linkage disequilibrium decay distance across the whole genome of the Maize1000 dataset.

**Figure S12.** The impact of different combinations of genetic and environmental features on model prediction accuracy. **a** The impact of different combinations of genetic and environmental features on model prediction accuracy of plant height in Maize2808 dataset. **b** The impact of different combinations of genetic and environmental features on model prediction accuracy of flowering time in Maize2808 dataset. **c** The impact of different combinations of genetic and environmental features on model prediction accuracy of ear weight in Maize2808 dataset. **d** The impact of different combinations of genetic and environmental features on model prediction accuracy of plant height in Wheat286 dataset. **e** The impact of different combinations of genetic and environmental features on model prediction accuracy of flowering time in Wheat286 dataset. **f** The impact of different combinations of genetic and environmental features on model prediction accuracy of yield in Wheat286 dataset. No_EPs represent no EPs were used, Raw_EPs represent raw environmental parameters were used, and RD_EPs represent dimensionality-reduced environmental parameters. All-SNPs: Genome wide quantitative trait nucleotides without feature extraction. Main-TAMs: environmental stability trait-associated markers. E-TAMs: Genotype-environment interactions trait-associated markers plus phenotypic plasticity trait-associated markers. All-TAMs: Main-TAMs plus E-TAMs. Random1: Based on the total number of TAMs identified by GWAS, SNPs were randomly selected from the genome. Subsequently, loci within 31.8 kb upstream and downstream of the randomly selected SNPs were extracted as genetic features for the Maize2808 dataset, while loci within 4 cM upstream and downstream were extracted for the Wheat286 dataset. Random2: Based on the number of All-TAMs and all SNPs within the intervals of 31.8 kb for the Maize2808 dataset and 4 cM for the Wheat286 dataset (upstream and downstream of the TAMs), an equivalent number of SNPs were randomly selected from the genome as genetic features.

**Figure S13.** Performance of automated machine learning-based genomic prediction across different prediction scenarios. Scenario 1: Phenotype prediction of untested genotypes in tested environments. Scenario 2: Phenotype prediction of tested genotypes in untested environments. Scenario 3: Phenotype prediction of untested genotypes in untested environments. **a** Prediction accuracy of plant height across different environments in the Maize2808 dataset. **b** Prediction accuracy of flowering time across different environments in the Maize2808 dataset. **c** Prediction accuracy of ear weight across different environments in the Maize2808 dataset. **d** Prediction accuracy of plant height across different environments in the Wheat286 dataset. **e** Prediction accuracy of flowering time across different environments in the Wheat286 dataset. **f** Prediction accuracy of yield across different environments in the Wheat286 dataset.

**Figure S14.** Multi-environment joint genomic prediction cross-environments and cross-genotypes using an automated machine learning model. **a, b** Prediction accuracy of plant height across different environments in the Maize366 and Maize5844 datasets, respectively. **c, d** Prediction accuracy of flowering time across different environments in the Maize366 and Maize5844 datasets, respectively. **e, f** Prediction accuracy of ear weight across different environments in the Maize366 and Maize5844 datasets, respectively.

**Leveraging** **Automated Machine Learning for** **Environmental Data-Driven Genetic Analysis and Genomic Prediction in Maize Hybrids**

Kunhui He^1,2,†^, Tingxi Yu^1,2,†^, Shang Gao^1,2^, Shoukun Chen^1,2^, Liang Li^1^, Xuecai Zhang^1,2,3^, Changling Huang^1,2^, Yunbi Xu^1^, Jiankang Wang^1,2^, Boddupalli M. Prasanna^4^, Sarah Hearne^3^, Xinhai Li^1^, Huihui Li^1,2*^

^1^ State Key Laboratory of Crop Gene Resources and Breeding, Institute of Crop Sciences, Chinese Academy of Agricultural Sciences (CAAS), CIMMYT-China office, Beijing, 100081, China.

^2^ Nanfan Research Institute, CAAS, Sanya, Hainan, 572024, China.

^3^ International Maize and Wheat Improvement Center (CIMMYT), Apdo. Postal 6-641, Texcoco, D.F. 06600, Mexico

^4^ CIMMYT, International Centre for Research in Agroforestry (ICRAF) House, Nairobi 00100, Kenya

^†^ These authors contributed equally to this article.

^*^ Corresponding author: Huihui Li, email: [lihuihui@caas.cn](mailto:lihuihui@caas.cn).

**Supporting Results**

To evaluate the performance of our framework across different maize hybrid populations and species, we utilized publicly available datasets from Xiao et al. (2021)^1^ for maize and Sukumaran et al. (2015)^2^ for wheat. The maize dataset comprised 8,652 hybrids, with phenotypic evaluations conducted for PH, FT, and ear weight (EW) across five locations in China over two growing seasons (2014 and 2015). These hybrids were partitioned into three datasets: Maize2808, Maize366, and Maize5844 (Table S11, Supporting Information). Maize2808 underwent phenotypic evaluation in 2014, Maize366, a subset of Maize2808, was evaluated in 2015, and Maize5844, which has no genotypic overlap with the other two datasets, was also evaluated in 2015. Maize2808 was used for GWAS analysis and genomic prediction model construction, while Maize366 was used to test cross-environment predictive ability, and Maize5844 was applied to evaluate cross-genotype and cross-environment predictive capabilities. The wheat dataset included 286 varieties (Wheat286), with PH, FT, and yield (YLD) evaluated over four growing seasons (from 2009 to 2012) at the CIMMYT experimental station near Ciudad Obregón, Sonora, Mexico (Table S12, Supporting Information).

We first established linear relationships between environmental parameters and traits in the Maize2808 and Wheat286 datasets, the latter including 11 developmental stages^3^, to identify critical development stage-environment windows, then calculated PP parameters for each genotype and trait based on these windows (Tables S13 and S14, Supporting Information). To search for TAMs that potentially influence PP and G×E interactions, we performed single-environment GWAS using the PP parameters and conducted multi-environment joint GWAS using trait values.

In Maize2808, GWAS identified 833 unique TAMs, including 440 PP-TAMs, 334 Main-TAMs, and 59 G×E-TAMs. Among these, 169 PP-TAMs, 142 Main-TAMs, and 13 G×E-TAMs were associated with PH; 138, 94, and 23 with FT; and 133, 98, and 23 with EW, respectively (Table S15, Supporting Information). In Wheat286, GWAS identified 81 unique TAMs, including 36 PP-TAMs, 33 Main-TAMs, and 12 G×E-TAMs. Among these, 14 PP-TAMs, 14 Main-TAMs, and 2 G×E-TAMs were associated with PH; 8, 12, and 3 with FT; and 14, 7, and 7 with YLD, respectively (Table S16, Supporting Information). Further analysis in Maize2808 revealed that 5.0% of the total PP-QTLs and 54.5% of the total G×E-QTLs colocalized for PH, 5.8% and 30.0% for FT, and 7.1% and 35.0% for EW (Table S17, Supporting Information). In Wheat286, 8.3% of the total PP-QTLs and 50.0% of the total G×E-QTLs colocalized for PH, 14.3% and 33.3% for FT, and 8.3% and 16.7% for YLD (Table S18, Supporting Information). In line with our above analyses in the Maize1000 dataset, these findings highlighted the differences in genetic basis between PP and G×E interactions.

We then explored the impact of various genetic and environmental features on model performance. In Maize2808, using All-TAMs for model training yielded higher predictive accuracy than using All-SNPs, Main-TAMs, or E-TAMs alone for all traits (Figure S12a-c, Supporting Information). Compared to All-SNPs, employing All-TAMs resulted in average increases of 3.35%, 5.40%, and 6.20% in predictive accuracy for PH, FT, and EW, respectively. Combining All-TAMs and DR_EPs as features achieved the highest predictive accuracy. Compared to All-SNPs, using both All-TAMs and DR_EPs increased predictive accuracy from 0.807 to 0.923 for PH (a 11.88% increase, percent change = (0.923–0.807)/0.807×100%), from 0.613 to 0.979 for FT (a 59.87% increase, percent change = (0.979–0.613)/0.613×100%), and from 0.428 to 0.828 for EW (a 93.20% increase, percent change = (0.828–0.428)/0.428×100%). Similar results were observed in Wheat286, wherein combining All-TAMs and DR_EPs also yielded the highest predictive accuracy (Figure S12d-f, Supporting Information). Compared to All-SNPs, using both All-TAMs and DR_EPs increased predictive accuracy from 0.532 to 0.753 for PH (a 41.59% increase, percent change = (0.753–0.532)/0.532×100%), from 0.316 to 0.938 for FT (a 196.69% increase, percent change = (0.938–0.316)/0.316×100%), and from 0.342 to 0.786 for YLD (a 129.84% increase, percent change = (0.786–0.342)/0.342×100%). These results confirmed the effectiveness of applying trait-associated genetic loci and DR_EPs as features to enhance predictive accuracy.

Using All-TAMs and DR_EPs as features, we employed our Auto-GS model for phenotypic predictions under three scenarios: Scenario 1) Phenotype prediction of untested genotypes in tested environments; Scenario 2) Phenotype prediction of tested genotypes in untested environments; and Scenario 3) Phenotype prediction of untested genotypes in untested environments. The model performed well across all scenarios. For instance, in Maize2808, the predictive accuracy for PH ranged from 0.814 to 0.863 for Scenario 1, 0.800 to 0.868 for Scenario 2, and 0.825 to 0.879 for Scenario 3 across different environments (Figure S13a, Supporting Information). For FT, the predictive accuracy ranged from 0.608 to 0.633 for Scenario 1, 0.517 to 0.607 for Scenario 2, and 0.608 to 0.645 for Scenario 3 across different environments (Figure S13b, Supporting Information). Similarly, for EW, the predictive accuracy ranged from 0.397 to 0.468 for Scenario 1, 0.253 to 0.390 for Scenario 2, and 0.399 to 0.475 for Scenario 3 across different environments (Figure S13c, Supporting Information).

In Wheat286, the predictive accuracy for PH ranged from 0.339 to 0.668 for Scenario 1, 0.411 to 0.822 for Scenario 2, and 0.374 to 0.734 for Scenario 3 across different environments (Figure S13d, Supporting Information). For FT, the predictive accuracy ranged from 0.359 to 0.497 for Scenario 1, 0.260 to 0.579 for Scenario 2, and 0.250 to 0.475 for Scenario 3 across different environments (Figure S13e, Supporting Information). For YLD, the predictive accuracy ranged from 0.254 to 0.467 for Scenario 1, 0.230 to 0.520 for Scenario 2, and 0.219 to 0.560 for Scenario 3 across different environments (Figure S13f, Supporting Information).

Additionally, we applied the model trained on Maize2808 to predict phenotypes for Maize366 and Maize5844. For PH, the predictive accuracies achieved were 0.675 to 0.871 for Maize366 and 0.271 to 0.323 for Maize5844 across different environments (Figure S14a,b, Supporting Information). For FT, the predictive accuracies were 0.186 to 0.709 for Maize366 and 0.096 to 0.260 for Maize5844 across different environments (Figure S14c,d, Supporting Information). For EW, the predictive accuracies were 0.164 to 0.592 for Maize366 and 0.095 to 0.217 for Maize5844 across different environments (Figure S14e,f, Supporting Information). These results further demonstrated the robust cross-environment predictive performance of our model.

**References**

1. Y. Xiao, S. Jiang, Q. Cheng, X. Wang, J. Yan, R. Zhang, F. Qiao, C. Ma, J. Luo, W. Li, H. Liu, W. Yang, W. Song, Y. Meng, M. L. Warburton, J. Zhao, X. Wang, J. Yan, *Genome Biol*. **2021**, *22*, 148.
2. S. Sukumaran, S. Dreisigacker, M. Lopes, P. Chavez, M. P. Reynolds, *Theor. Appl. Genet*. **2015**, *128*, 353.
3. J. White, J. Edwards. *Wheat growth and develop.* **2008**, p. 41.
